# Paroxysmal hemicrania masquerading as a stroke in an elderly gentleman: case report

**DOI:** 10.1186/s12877-020-01768-5

**Published:** 2020-10-07

**Authors:** Boon Hian Tan, Astrid Melani Suantio, Yeow Hoay Koh

**Affiliations:** 1 Department of General Medicine, Geriatric Medicine, Sengkang General Hospital, 110 Sengkang East Way, Singapore, 544886 Singapore; 2 Department of Neurology, National Neuroscience Institute, Singapore General Hospital, Outram Road, Singapore, 169608 Singapore

**Keywords:** Paroxysmal hemicrania, Stroke mimics, Horner’s syndrome, Trigeminal autonomic cephalalgia, TAC, Case report

## Abstract

**Background:**

Paroxysmal hemicrania has not been associated with ipsilateral weakness, loss of sensation and Horner’s syndrome. This report is the first of its kind documented in literature.

**Case presentation:**

This was an elderly, sixty-five-year-old Chinese male who presented with a headache fulfilling criteria of paroxysmal hemicrania and was found to have signs of ipsilateral conjunctival injection, Horner’s syndrome, weakness and loss of sensation; with resolution of the patient’s physical signs after relief of the headache. Brain magnetic resonance imaging did not show any strokes or other headache mimics. The patient had a marked response to indomethacin and a decrease of headache intensity and frequency with indomethacin prophylaxis.

**Conclusions:**

Paroxysmal hemicrania has joined the list of stroke chameleons and that it would be one of the differentials in a patient with hemiplegia, hemisensory loss, autonomic signs and severe headache. It suggests that paroxysmal hemicrania in the elderly present atypically.

## Background

Trigeminal autonomic cephalalgia (TAC) is a diverse group of unilateral headaches with ipsilateral autonomic features [1]. The paroxysmal hemicrania (PH) subtype occurs more commonly in females and typically affects patients aged between 40 and 50 years of age [2–4]. It is a disease involving multiple episodes of strictly unilateral, severe, short-lasting headaches occurring with cranial autonomic features with good response to indomethacin [1, 3, 4]. It is uncommon, but not unheard of, for elderly patients of age of 65 years and above, to also be affected [3, 4]. There have been reports of patients as old as 81 years of age with PH [4].

The physical examination of patients known to have PH has had reports of loss of sensation to the ipsilateral face, loss of visual acuity and the acquisition of a relative afferent papillary defect [3, 4]. The loss of motor power in the limbs and the development of Horner’s syndrome during a PH episode have not yet been described in the current literature [3, 4]. There are many diseases that are initially diagnosed as PH and subsequently revised, based on subsequent neuroimaging evidence, as other diagnoses. These includes strokes involving the brainstem, arterio-venous malformations, meningiomas, bulking pituitary lesions, vascular loops encompassing the trigeminal nerve and Moya-Moya disease [4–13]. Strokes are known to be more common in the geriatric population, whom constitute 53.4% of all adult strokes [14]. This is a case report of a stroke mimics in an elderly male patient, of which the clinical progression, investigations and clinical response have led to the diagnosis of paroxysmal hemicrania; and is one of the first reports of a PH masquerading as a stroke.

## Case presentation

### Demographics and past medical history

This was 65-year-old Asian man, independent with instrumental activities of daily living. He was a non-smoker and a teetotaller. He was diagnosed 1 year ago with cervical spondylosis with no residual deficits. He did not have a history of headaches nor a history of psychiatric disorders.

### First admission to hospital (Dec 2018)

The patient initially presented in December 2018 to our local hospital with symptoms of headache, left hemiparesis and left hemisensory deficit occurring intermittently for 3 days. There was transient diplopia on downward gaze. These symptoms would fluctuate during the day. There were no fevers, head injuries, seizures, nor neck trauma.

The patient’s vital signs on admission showed a blood pressure (BP) of 115/64 mmHg with a regular heart rate (HR) of 68 beats per minute (bpm). He was apyrexial throughout admission. Physical examination showed a new onset left-sided facial droop with left-sided upper limb weakness of Medical Research Council (MRC) grade 4 out of 5 and reduced sensation to pinprick over the left face, upper and lower limbs. Both eyes were unable to abduct fully and developed compensatory bidirectional nystagmus during abduction. The remaining cranial nerves examination showed no other deficits. There were no cortical signs - there was no visual field loss, no aphasia, no dysarthria, no dysphasia nor hemineglect. His tone and reflexes were normal and the Babinski’s reflexes were normal bilaterally.

**Fig. 1:**
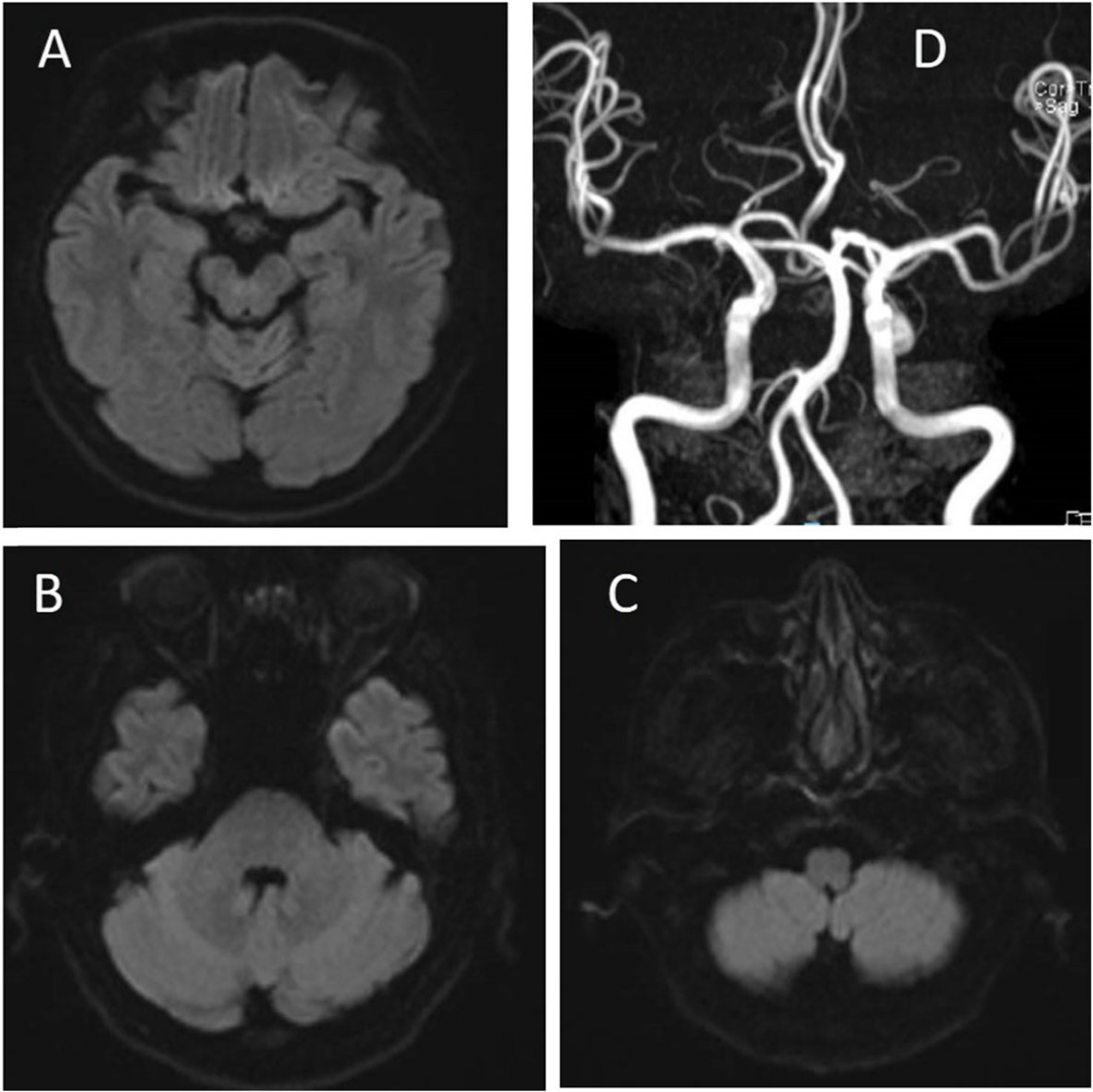
Magnetic resonance imaging of the brain showing lack of diffused weighted hyperintensities of (**a**) midbrain (**b**) pons (**c**) medulla. (**d**) normal magnetic resonance angiography

An urgent computerised tomographic (CT) scan of the brain showed no intracranial haemorrhage nor infarction. He was started on aspirin and clopidogrel for secondary stroke prevention. His Magnetic Resonance Imaging (MRI) of the brain did not show any restricted diffusion to indicate an acute infarct; with neither his Magnetic Resonance Angiography (MRA) nor his carotid ultrasound showing any flow-limiting stenoses, occlusions nor aneurysms (Fig. 1). The echocardiogram showed an ejection fraction of 57% and normal sized atria and otherwise unremarkable valves. There were neither intracardiac thrombi nor valvular vegetations. A 24-h telemetry monitoring via a Holter did not reveal any atrial fibrillation. His serum glucose, sodium potassium and corrected serum calcium tests, were all within normal limits.

One day after the presentation of his illness, his headache and neurological signs simultaneously resolved. His motor power had normalized to MRC grade 5/5, his sensation was restored and his nystagmus had disappeared. As his symptoms persisted beyond 24 h, he was given a diagnosis of an MRI-negative stroke and was continued on aspirin and clopidogrel for 6 weeks with lifelong clopidogrel thereafter. He was discharged well and without any new nor worsening neurological signs. Post-discharge, he was reviewed in the neurology clinic 3 months later. There was no new nor residual neurological deficits.

### Second admission to hospital one year later (Jan 2020)

**Fig. 2:**
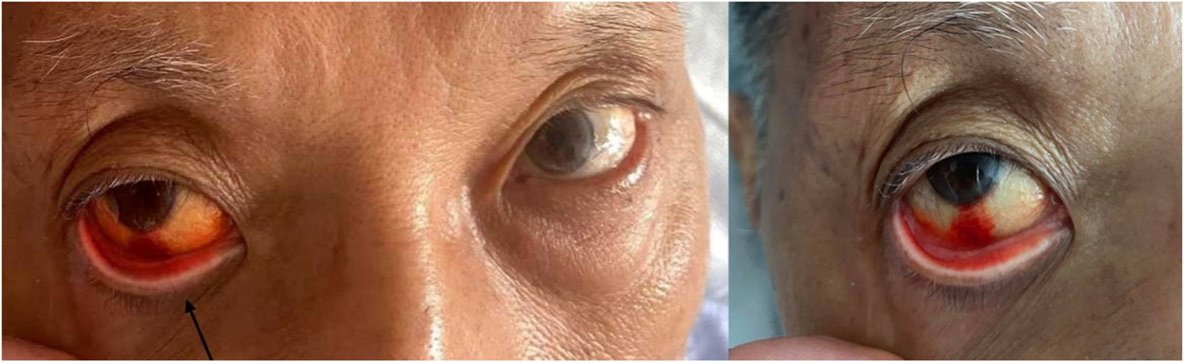
Conjunctival injection of the eye (solid arrow)

One year after the original event, the same patient again presented with sudden onset of a right-sided headache involving the entire right side of the head, with a radiation to the right orbit and the right neck. One hour after the onset of symptoms, the patient developed autonomic symptoms of right eye redness, right eye lacrimation and sweating over the right scalp. The severity of the headache was 9 out of 10 on a Likert scale, with each episode lasting 5–10 min before weaning off and then recurring again every 30–60 min for at least 12 h, with at least 20 attacks over 12 h. It was associated with diplopia and drooping of right eyelid, with right-sided upper limb and lower limb weakness. There was no antecedent head nor neck trauma, no fevers, no photophobia, no seizures nor syncopal episodes. He had been compliant to clopidogrel since the previous admission. Upon arrival in the hospital, the symptoms of headache and weakness remained and his eyes were still found to be injected (Fig. 2).

His usual frequency of headaches was nearly daily for the past year which came in waves of headaches lasting 10–20 min for a total duration of 2–3 h, with symptoms of nasal congestion, rhinorrhoea, bilateral eyelid swelling and sweating.

**Fig. 3:**
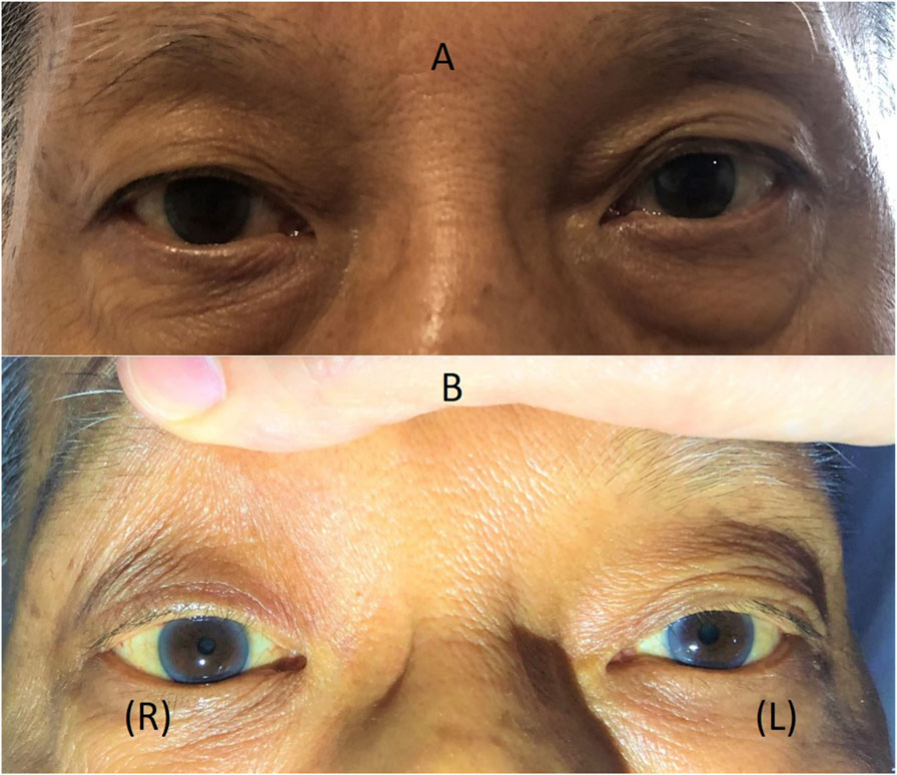
Horner’s syndrome. (**a**) mild ptosis on right eye with slight elevation of right lower eyelid (**b**) anisocoria with right pupil size (R) smaller than left pupil size (L)

During physical examination, his BP was 119/67 mmHg, his HR was 64 bpm and regular. He remained apyrexial throughout admission. He was found to have a right partial ptosis with anisocoria. The right conjunctiva was injected. His right pupil was 2 mm and left pupil was 3 mm, and there was a slight elevation of his right lower eyelid with delayed pupillary dilatation in dim light (Fig. 3). He had binocular diplopia without ophthalmoplegia at bilateral lateral gaze without any nystagmus. There was no skew deviation of the eyes. He was dysarthric. He had decreased motor power of MRC grade 4 out of 5 involving the entire right upper and lower limbs. There was a right-sided facial droop; with a loss of sensation over the right side of the face, the entire upper and lower limbs to modalities of pinprick, temperature and fine touch. The tongue was central with equal movement of the uvulae. His tone was decreased over the right upper and lower limbs. There was no pronator drift and the Babinski’s reflexes were plantar bilaterally.

**Fig. 4:**
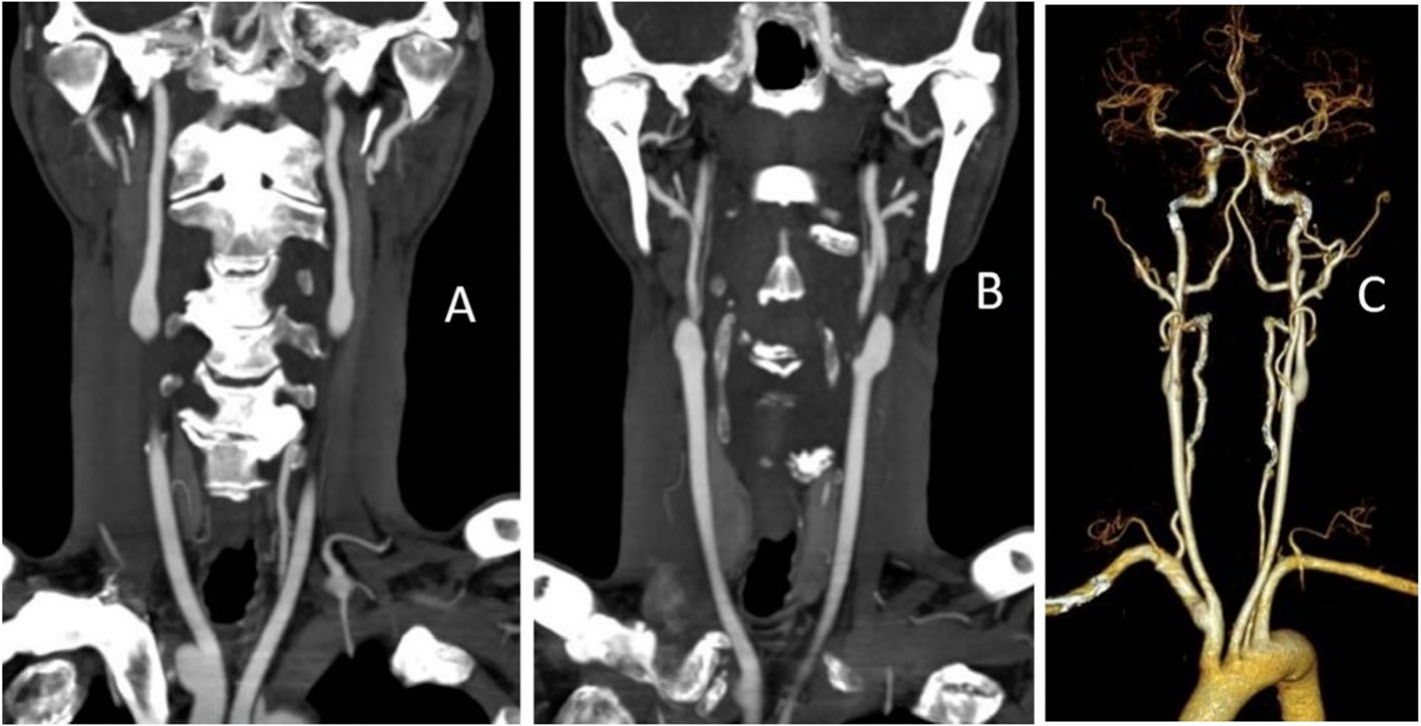
Computerized tomography angiogram showing (**a**, **b**) patency and lack of dissection in the carotids. (**c**) 3-dimensional reconstructed image of the same computerized tomography angiogram

The patient was placed back on aspirin and clopidogrel as his CYP2C19 genome tests showed that he was a normal metabolizer of clopidogrel. An urgent CT angiography (CTA) from the arch of the aorta to the Circle of Willis did not reveal any dissections, aneurysms and arteriovenous malformations, nor findings consistent with temporal arteritis (Fig. 4). No cranial infarction nor intracranial haemorrhage was seen at that time. He had persistent neurological deficits after the CT angiogram. His electrocardiogram showed normal sinus rhythm with neither evidence of acute coronary syndrome, atrial fibrillation, nor arrhythmias. His serum sodium, potassium and white blood cell counts were all within normal limits. A serum erythrocyte sedimentation rate was not performed as there was no symptoms of jaw claudication nor polymyalgia rheumatica; the patient’s blood haemoglobin was 13.2 g/dL.

The patient’s headache improved in the morning of the next day, albeit not completely resolved. His pain score was 4 out of 10 on a Likert scale. While the sensory loss had resolved, he had still a MRC power of 4 out of 5 in the upper and lower limbs. By midday 5 hours after, his headache had resolved and with it, his neurological deficits.

**Fig. 5:**
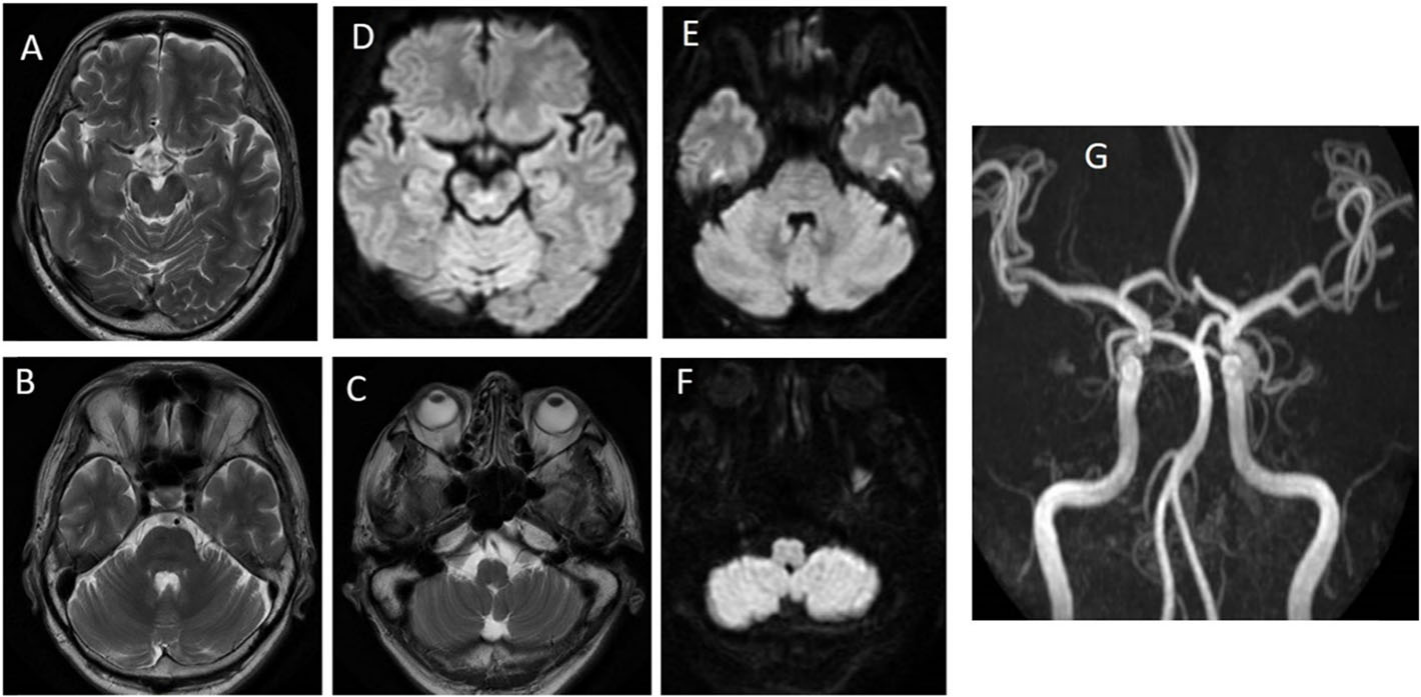
Magnetic resonance imaging of the brain showing T2 weighted images of (**a**) midbrain (**b**) pons (**c**) medulla and diffusion weighted images of (**d**) midbrain (**e**) pons (**f**) medulla. (**g**) Magnetic resonance angiogram

The duration from onset of disease to the resolution of symptoms had exceeded 24 h. An MRI with MRA after the complete resolution of neurological deficits did not show any restricted diffusion to indicate an acute infarct. The T2 weighted images did not show any hyperintensities suggestive of previous infarction. His MRA did not show any flow-limiting stenoses, occlusions, aneurysms or neurovascular conflicts with the trigeminal nerve (Fig. 5). There was no sinusitis in the CT nor MRIs performed. In view of a headache with autonomic symptoms, its duration and its frequency of symptoms, and the prompt resolution of its stroke-like signs when the headache resolved; the patient was given a diagnosis of probable paroxysmal hemicrania presenting as a stroke mimic and was subsequently started on indomethacin. As his symptoms resolved after indomethacin was started, we were not able to demonstrate a positive indomethacin test during that admission. He had discharged well without any recurrence of symptoms. A subsequent review in clinic showed that he had a decreased frequency of headaches from that of a daily headache to that of one to two times a week, with significantly less pain during each episode since he started taking indomethacin at a dose of 50 mg every night. During a headache episode, indomethacin would take an hour to act and its effects lasted for the whole night after abortion of headache. His screening 15-point Geriatric Depression Scale was 2 and his 28-item Chinese Mini Mental state exam was 26. There were no side effects of gastrointestinal bleeding during his follow up.

## Discussion and conclusion

We have put forward a probable diagnosis of PH, and it is supported with evidence of at least 20 attacks within 12 h, unilateral features of orbital pain, conjunctival injection, lacrimation, ptosis and miosis. There is a response to indomethacin with marked improvement of symptoms upon ingestion of indomethacin with sustained response.

The main differential diagnosis of headache in our patient is that of a hemiplegic migraine under the subtypes of brainstem, sensory and motor migraine. Firstly, the duration of pain attacks is uniformly less than 4 h per episode, whether occurring inpatient or as outpatient; compared to the duration of pain of migraines which usually last 4–72 h [1]. Secondly, his non-motor symptoms of diplopia, dysarthria and sensory loss all lasted more 3 h and is not consistent with the International Classification of Headache Disorders 3rd edition (ICHD-3) classification criteria. There were no visual auras in our patient and 90% of all migraine auras are visual [1], and additionally brainstem migraines are also rare, occurring for approximately 10% of all migraines [15]; making a diagnosis of migraine unlikely. Lastly neither ptosis, miosis nor autonomic symptoms have ever been described as migraine auras in ICHD-3 [1] nor in a review of brainstem migraines (Table 2 in reference [15]). Another differential diagnosis is that of a migrainous infarct [1], which is unlikely as this diagnosis is ruled out with an absence of restricted diffusion in an MRI performed during the acute episode of stroke. While there is a proportion of posterior circulation infarcts which are not detected on neuroimaging during the acute event, our patient had a MRI one year following the initial attack in 2019 and there was no evidence of T2 hyperintensities suggestive of a previous infarct. A less likely differential could be that the patient was having multiple episodes of transient ischaemic attacks, of which is unlikely given that his symptoms and signs were always related to headaches, and went away when the headache resolved, and that there was a paucity of vascular disease in the T2 as well as a normal MRA in the MRI performed in the second admission.

This case report suggests that PH may present unusually as a stroke mimic. The index of suspicion of a stroke in both presentations were high. In the first presentation, the patient belonged to the geriatric age group and presented signs of weakness and nystagmus, which were not typically related to the autonomic nervous system. In the second presentation, he was previously thought to have had a DWI-negative stroke and had motor signs developing in limbs contralateral to the previous episode.

While there have been accounts of Horner’s syndrome with paradoxical ipsilateral sweating, these cases are often found in post-surgical patients with following cervical sympathectomy with resultant anomalous vagal connections to the postganglionic sympathetic nerves [16]. The Horner’s syndrome that the patient developed can thus be considered as atypical in its presentation and can be a clue to the diagnosis of PH which shares examination signs of ptosis and miosis.

Our patient has responded to indomethacin treatment as well as improvement of frequency and severity of headache attacks with low dose indomethacin prophylaxis 50 mg every night. This is consistent with current treatment recommendations of which further follow-up is suggested, in view of gastrointestinal toxicity of indomethacin and to taper mediations to the lowest possible dose [17]. Additionally, as the pathophysiology of PH is not fully described [17, 18], this report may help guide further research in this area and in particular, PH and its pathophysiology in relation to the brainstem. In summary, the learning point is that PH has joined the list of stroke chameleons and that it would be one of the differentials in a patient with hemiplegia, hemisensory loss, autonomic signs and severe headache. However, as many headaches are actually brainstem infarcts or dissections, the safest option would be to undergo neuroimaging first and to manage the patient assuming there was a stroke.

## Data Availability

Not applicable.

## Abbreviations

BP: Blood Pressure
bpm: Beats Per Minute
CT: Computerized Tomographic
CTA: Computerized Tomographic Angiogram
HR: Heart Rate
ICHD-3: The International Classification of Headache Disorders 3rd edition
MRC: Medical Research Council
MRI: Magnetic Resonance Imaging
MRA: Magnetic Resonance Angiography
PH: Paroxysmal Hemicrania
T2: Transverse Relaxation Time
TAC: Trigeminal Autonomic Cephalalgia
